# Behavioral Changes in a Pediatric Patient With Sotos Syndrome: A Case Emphasizing the Importance of Coordinated Care

**DOI:** 10.7759/cureus.66093

**Published:** 2024-08-03

**Authors:** Tristan Hichkad, Emma E Guld, Gabriella Assi

**Affiliations:** 1 Research, Lake Erie College of Osteopathic Medicine, Bradenton, USA; 2 Pediatrics, Lake Erie College of Osteopathic Medicine, Bradenton, USA; 3 Pediatrics, Children's Health Associates, Jacksonville, USA

**Keywords:** diabetes mellitus type 2, multidisciplinary treatments, central precocious puberty, overgrowth syndrome, sotos syndrome

## Abstract

Sotos syndrome is a rare overgrowth condition characterized by tall stature, distinctive facial features, and learning disabilities. It is primarily caused by a microdeletion of the nuclear receptor-binding set domain protein 1 (NSD1) gene on chromosome 5q35. Patients often present with various clinical manifestations, including tall stature, precocious puberty, cardiac anomalies, and mild intellectual disability. Management of Sotos syndrome involves a multidisciplinary approach due to its complex nature and potential comorbidities. This case discusses the management of a 10-year-old female with a known gene mutation consistent with Sotos syndrome that presented to the clinic with behavioral changes, and highlights the importance of integrated care models when addressing complex clinical scenarios.

## Introduction

Sotos syndrome is a rare disorder caused by a microdeletion of nuclear receptor-binding set domain protein 1 (NSD1) found on 5q35. The prevalence is estimated at 1 in 14,000, with a significant majority (over 95% of cases) resulting from de novo mutations [1]. Common clinical manifestations include tall stature, advanced bone age, precocious puberty, frontal bossing, dolichocephaly, a high-arched palate, and hypotonia. Additional findings include neonatal jaundice, cardiac anomalies, renal anomalies, joint laxity, and scoliosis [1,2]. A vast majority of individuals with Sotos syndrome also have a mild intellectual disability and at some point in time may present with behavioral changes, primarily aggression and tantrums [3,4]. When these syndromic features are present at birth or young age, Sotos syndrome may be suspected and warrant genetic testing if more common conditions have been ruled out.

In general, Sotos syndrome has a fairly good prognosis. Children with Sotos syndrome have been reported to continue into adulthood with a normal life expectancy, despite there being a wide spectrum of dependency [5]. Though considered a pediatric condition, limited data have been reported describing features in adults. Furthermore, current literature is limited on how Sotos syndrome may affect individuals' health and well-being into adulthood. Our patient has many comorbidities at a young age, such as type two diabetes mellitus (T2DM), learning disabilities, and anxiety, which has created a unique profile that has not been fully specified in current literature. This case created a multi-layered challenge for both the patient, their caretaker, and the clinician. Our case describes this unique presentation of Sotos syndrome and how the use of a multidisciplinary approach may be utilized in its management.

## Case presentation

A 10-year-old female, accompanied by her mother, presented to the pediatric clinic due to recent increased anger and aggression. The patient admitted to breaking items at school and home without specific triggers, mentioning anxiety around attending school due to discomfort in crowded settings. Her preference for limited social interaction both at school and home was noted. Her medical history included overgrowth syndrome, idiopathic central precocious puberty, mild scoliosis, and T2DM. Current medications included metformin and Bydureon, and vaccinations were up to date. Vital signs were normal, with a height of 6 ft 0 in (>97th percentile), weight at 260 lb (99th percentile), and a BMI exceeding the 99th percentile. Previous genetic testing diagnosed the patient's overgrowth as Sotos syndrome.

Physical examination revealed tall stature, macrocephaly (head circumference >97th percentile), frontal bossing, and mild hypotonia. Acanthosis nigricans, a common sign of insulin resistance, was present along the skin creases of the neck. Sexual maturity rating was 5 in the breasts and 4 in the pubic region. The remaining exam was unremarkable. At this time, the assessment given was Sotos syndrome, an unspecified anxiety disorder, T2DM, idiopathic central precocious puberty, and mild scoliosis.

The patient’s management plan involves referrals to behavioral counselors, reevaluation of her individualized educational program (IEP) with the school counselor, a nutritionist for T2DM dietary modifications, endocrinology follow-up, and orthopedic follow-up (Table 1). The patient’s mother was given a list of behavioral counselors and educational support. It was then recommended that the patient receive a psychiatric evaluation due to the severity of the effects of Sotos syndrome on physical and mental growth and development. The patient's previous developmental delay, degree of intellectual disability, and new behavioral changes would be addressed during the psychiatric consultation. The patient’s mother stated that she had already scheduled a follow-up with an endocrinologist to monitor the progression of the patient’s T2DM and adjust medications as needed, and a referral to a nutritionist for dietary modifications in regard to her T2DM was placed. A follow-up appointment with orthopedics was ordered to reevaluate her scoliosis.

**Table 1:** Clinical evaluation of patients with suspected Sotos syndrome SD: standard deviation; NSD1: nuclear receptor-binding set domain protein 1

Evaluation of Sotos Syndrome	
Clinical manifestations	Distinctive facial features (prominent forehead, down slanting palpebral fissures, long and narrow face), developmental delay, overgrowth (height and/or head circumference >2 SD above mean)
Diagnosis	Genetic testing for deletion of NSD1 (specific), radiographs showing bone age > chronological age (nonspecific)
Management	Treat manifestations → referral to appropriate specialists (e.g. speech/language pathology, psychiatry, endocrinologist, orthopedics, etc.)

## Discussion

**Figure 1:**
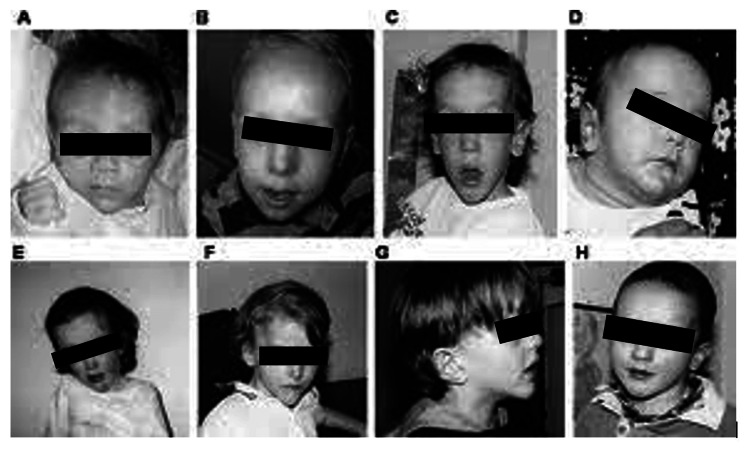
The facial gestalt of children with Sotos syndrome. Note the prominent facial features including macrocephaly (B, D), frontal bossing (B, D, H), and pointed chin (C, F, G). Source: Leventopoulos et al., 2009. doi: 10.1016/j.pediatrneurol.2008.11.013 [2]. Permission is obtained from the original publisher.

Sotos syndrome can manifest with a wide array of complex physical and mental comorbidities, which may pose quite a challenge to the clinician. Figure 1 presents facial features observed in other cases of Sotos syndrome, some of which were able to match the facial appearance of our patient which further supported the established diagnosis. The diagnosis of T2DM introduces the need for pharmacologic management, dietary regulation, and lifestyle modifications. The excess growth and scoliosis necessitate observation by orthopedic specialists. The new onset of aggressive behavior and feelings of anxiety require special attention and close monitoring. With many physical and mental problems to address in this patient’s case, mainstay pharmaceutical and symptomatic treatment alone will have limited effectiveness in completely resolving this disorder [6]. Children with Sotos syndrome experience vast differences in growth patterns, puberty, and reproduction, and new and evolving medical problems are nearly always observed. Varying degrees of intellectual disability and behavioral issues were reported in children evaluated with Sotos syndrome, which may necessitate some level of school accommodations and specialized education programs [5]. These circumstances warrant the use of evidence-based integrative models that will better address real-world challenges of combined physical and mental conditions [6].

Foster et al. [5] have helped to clarify some clinical features that may persist throughout adulthood in individuals with Sotos syndrome, such as persistent facial features and degree of intellectual disability. It remains unclear whether Sotos syndrome affects the severity of associated medical conditions or whether they are independent factors. Nonetheless, a model of integrated care, which has been shown to enable access to services and improve patient satisfaction, can be applied to patients in order to achieve the best outcomes [7].

Our patient's plan integrates conventional pharmacological treatments with novel adjunctive treatments where applicable. Most importantly, the chief complaint of behavioral changes was addressed since a vast majority of individuals with a mental health disorder do not receive treatment [4]. From a clinical practice perspective, this case underscores the importance of a holistic approach to patient care. Clinicians should be aware of the potential for new and existing conditions in patients with Sotos syndrome and be prepared to coordinate care across multiple specialties. The management of this particular case required the collaboration of various healthcare professionals, including endocrinologists, mental health professionals, and educational specialists. A multidisciplinary approach should always be considered when managing complex cases in clinical practice.

## Conclusions

This case report provides a comprehensive review of a patient found to have Sotos syndrome with concurrent T2DM, developmental delay, constitutional overgrowth, and presenting with behavioral changes in early adolescence. The co-occurrence of these conditions presents unique challenges and underscores the importance of a multidisciplinary approach to patient care. Despite the sparse number of reported cases, children who progress into adulthood can continue to be followed to observe the manifestations and allow a better understanding of individuals’ course of life. Further research will be needed to be able to explain whether Sotos syndrome has any impact on individuals' risk of acquiring T2DM and how severe the disease may progress. Future investigations should also aim to understand what behavioral changes clinicians can expect particularly in children with specific genetic disorders. Maintaining a high index of suspicion for these changes can assist in timely referrals, which can assist in optimal care and prevent any potential complications into adulthood. By improving our understanding of Sotos syndrome and its associated comorbidities, we can develop more effective treatment strategies and set children up for a healthy life with longevity.

## References

[1] Manor J, Lalani SR (2020). Overgrowth syndromes-evaluation, diagnosis, and management. Front Pediatr.

[2] Leventopoulos G, Kitsiou-Tzeli S, Kritikos K, Psoni S, Mavrou A, Kanavakis E, Fryssira H (2009). A clinical study of Sotos syndrome patients with review of the literature. Pediatr Neurol.

[3] Lane C, Milne E, Freeth M (2016). Cognition and behaviour in Sotos syndrome: a systematic review. PLoS One.

[4] Tatton-Brown K, Cole TRP, Rahman N (2022). Sotos syndrome. GeneReviews® [Internet].

[5] Foster A, Zachariou A, Loveday C (2019). The phenotype of Sotos syndrome in adulthood: a review of 44 individuals. Am J Med Genet C Semin Med Genet.

[6] Ee C, Lake J, Firth J (2020). An integrative collaborative care model for people with mental illness and physical comorbidities. Int J Ment Health Syst.

[7] Baxter S, Johnson M, Chambers D, Sutton A, Goyder E, Booth A (2018). The effects of integrated care: a systematic review of UK and international evidence. BMC Health Serv Res.

